# Antihypertriglyceridemia and Anti-Inflammatory Activities of *Monascus*-Fermented Dioscorea in Streptozotocin-Induced Diabetic Rats

**DOI:** 10.1155/2011/710635

**Published:** 2011-05-11

**Authors:** Yeu-Ching Shi, Jiunn-Wang Liao, Tzu-Ming Pan

**Affiliations:** ^1^Department of Biochemical Science and Technology, College of Life Science, National Taiwan University, No. 1, Sec. 4, Roosevelt Road, Taipei 10617, Taiwan; ^2^Institute of Veterinary Pathobiology, College of Veterinary Medicine, National Chung Hsing University, Taichung 402, Taiwan

## Abstract

The rice fermented by *Monascus*, called red mold rice (RMR), and has a long tradition in East Asia as a dietary staple. *Monascus*-fermented dioscorea called red mold dioscorea (RMD) contains various metabolites to perform the ability of reducing oxidative stress and anti-inflammatory response. We used Wistar rats and induced diabetes by injecting streptozotocin (STZ, 65 mg/kg i.p.). RMD was administered daily starting six weeks after disease onset. Throughout the experimental period, significantly (*P* < .05) lowered plasma glucose, triglyceride, cholesterol, free fatty acid and low density lipoprotein levels were observed in the RMD-treated groups. The RMD-treated diabetic rats showed higher activities of glutathione disulfide reductase, glutathione reductase, catalase and superoxide dismutase (*P* < .05) in the pancreas compared with the diabetic control rats. RMD also inhibited diabetes-induced elevation in the levels of interleukin (IL)-1*β*, IL-6, interferon-*γ* and tumor necrosis factor-*α*. Pancreatic *β*-cells damaged by STZ in the RMD supplemented groups were ameliorated. The results of this study clearly demonstrated that RMD possesses several treatment-oriented properties, including the control of hyperglycemia, antioxidant effects, pancreatic *β*-cell protection and anti-inflammatory effects. Considering these observations, it appears that RMD may be a useful supplement to delay the development of diabetes and its complications.

## 1. Introduction

Diabetes mellitus is a metabolic disease resulting from insulin deficiency, leading to high blood glucose levels and hyperglycemia [1]. Hyperglycemia resulting from defection in insulin action or insulin production leads to a number of complications [2]. The biochemical parameters changes such as formation of advanced glycation end products (AGEs) have increased the expression of proinflammatory cytokine genes [3]. Diabetic complications have demonstrated that the production of excess reactive oxygen species (ROS) leads to tissue injury or apoptosis [4]. The reduction of antioxidant enzyme activities and tissue glutathione (GSH) levels have been reported in diabetes mellitus [3]. Both oxidative stress and inflammation play a major role in the development of tissue insulin resistance [5].

Streptozotocin (STZ) is the most commonly used chemical to induce experimental diabetes in animals. Glucose overload may damage the cells through oxidative stress and inflammation, and oxidative stress has been suggested to be a contributory factor in the pathogenesis of diabetes [6, 7]. Hyperglycemia increases the inflammatory markers' tumor necrosis factor (TNF)-*α*, interleukin (IL)-1, and IL-6 [8, 9]. Overproduced proinflammatory cytokines enhances inflammatory stress in diabetes and diabetic complications. A great deal of evidence indicates close ties between inflammatory and metabolic pathways [10–12]. 

The solid state fermentation of rice by *Monascus*, called red mold rice (RMR), has a long tradition in East Asia as a dietary staple and food additive, which improves the digestion and revitalizes the blood [13, 14]. Monacolin K (lovastatin), an inhibitor of 3-hydroxy-3-methyglutaryl coenzyme A (HMG-CoA) reductase, is a worthy secondary metabolite found in RMR to inhibit the biosynthesis of cholesterol for treating hyperlipidemia [15]. RMR has been proven to improve the memory and learning ability in A*β*-infused rats, reduces the tumor progression of Lewis lung carcinoma cells and prevents the development of obesity [16–19]. RMR contains various metabolites, including dimerumic acid, tannin, phenol, and azaphilones [20], to perform the ability of antioxidative stress and anti-inflammatory response [20, 21]. 

Yam tubers of *Dioscorea* genus have been used as traditional medical herb substances [22], health food supplements and functional foods, for the treatment of diabetes in China [23]. Yam tubers have a multiple biological effect, including reducing blood glucose [24], lowering blood lipids [25], promoting an antitumor effect [26], and possessing antioxidant properties [27]. The presence of various phenolic compounds has been reported in yam tuber tissue, such as (+)-catechin [28] and cinnamic compounds [29].

According to the authors' knowledge, there has been no detailed study carried out to investigate the efficacy of red mold dioscorea (RMD) in the antioxidative and anti-inflammatory activity associated with diabetes in experiments on animals. This study determined the content of phenolic acids from RMD and examined antidiabetic, antioxidative, and anti-inflammatory effects in STZ-induced diabetic rats. The aim of the present study was to evaluate the chemoprevention of RMD in a diabetic animal model. The cytokine and antioxidation enzyme profile of STZ responses in the tissue were further analyzed.

## 2. Materials and Methods

### 2.1. Sample Preparation

*Monascus purpureus* NTU 568 was maintained on potato dextrose agar (PDA) slant at 4°C and transferred monthly. The preparation of red mold dioscorea (RMD) was carried out using the substrates of dioscorea (*Dioscorea batatas* Dence) root. Solid-state culture method was used to prepare the fermented contents [16]. At the end of cultivation, the crushed and dried products with the mold were used for the experiments.

### 2.2. Analysis of Phenolic Compounds

 Total polyphenolic compounds were extracted from 0.5 g of red mold dioscorea with 5 mL of methanol/1.5 N HCl (85/15, v/v) for 1 hr at ambient temperature (27–30°C). The suspension was centrifuged at 7,000 × g for 5 min. The supernatant was filtered through 0.45 *μ*m pore size filter, and 100 *μ*L was injected onto the high performance liquid chromatography (HPLC) system [30]. HPLC analyses were operated by an HPLC system (Agilent 1100 series, Santa Clara, CA, USA) and carried out on a Mightysil RP-18 GP column (250 × 4.6 mm, Kanto Chemical, Tokyo, Japan). The assay method and solvent system were derived from that of Akissoe et al. [30]. Commercial phenolic standards were characterized by their retention times and coinjected with red mold dioscorea extracts to identify the peaks.

### 2.3. Animals and Experimental Design

 Male Wistar rats, weighting 140–160 g, aged 5-6 weeks, were used for this experiment. The experiments were carried out in a qualified animal breeding room in the Animal Center at our institute (protocol complied with guidelines described in the “Animal Protection Law”, amended on Jan. 17, 2001 Hua-Zong-(1)-Yi-Tzi-9000007530, Council of Agriculture, Executive Yuan, Taiwan, ROC). Diabetes were induced by injecting intraperitoneally with 65 mg/kg streptozotocin (STZ, Sigma, St Louis, MO, USA) in 0.1 M acetate buffer and 230 mg/kg nicotinamide after fasting for 12 to 24 hrs. Rats with plasma glucose concentration of 200 mg/dL or greater were considered as diabetic animals and used in this study. The animals were randomly divided into six groups and each group contained six animals. Age-matched normal rats served as normal control (NC). Diabetic rats were divided into five groups: group 1: diabetic control (DC); group 2 received dioscorea 200 mg/kg (DM (diabetes mellitus) + 1X D); group 3 received red mold dioscorea 100 mg/kg (DM + 0.5X RMD); group 4 received RMD 200 mg/kg (DM + 1X RMD); group 5 received RMD 1,000 mg/kg (DM + 5X RMD). Rats were anaesthetized and sacrificed at the end of the 6-week treatment; the pancreas, spleen, and kidney were excised and washed thoroughly to clear off blood.

### 2.4. Biochemical Analysis

 Blood glucose, triglyceride (TG), total cholesterol (TC), high density lipoprotein cholesterol (HDL-C), low density lipoprotein cholesterol (LDL-C), serum insulin, serum-free fatty acid (FFA), and total protein were measured at 6 weeks of red mold dioscorea treatment. Blood glucose was immediately determined by the glucose oxidase method, using an analyzer [31]. Serum insulin was measured using an enzyme-linked immunosorbent assay (ELISA) insulin kit (Mercodia AB, Uppsala, Sweden). Plasma TG, TC, HDL-C, LDL-C, FFA, and total protein levels were measured in triplicate using an automatic biochemical analyzer (Beckman-700, Fullerton, CA, USA).

### 2.5. Antioxidant Enzymes Activities

 Following the termination of the animals, pancreas was immediately removed and homogenized with ice-cold 0.1 N Tris-HCl buffer (pH 7.4) (1 : 10, w/v). After centrifugation at 2,500 × g for 30 min at 4°C, the supernatant was collected for the measurement of antioxidant enzymes activities. The analysis of level of reactive oxygen species (ROS), activity of glutathione peroxidase (GPx), activity of glutathione reductase (GR), and activity of catalase (CAT) was carried out according to the methods previously reported [32–35]. The activity of superoxide dismutase (SOD) was determined by commercially available kit (Cayman, Ann Arbor, MI, USA).

### 2.6. Cytokine Analyses

 Tissues were immediately removed and homogenized with ice-cold 10 mM Tris-HCl buffer (pH 7.4) [36]. After centrifugation at 9,000 × g for 30 min at 4°C, the supernatant was collected for the measurement of cytokines. The levels of IL-1*β*, TNF-*α*, IL-2, IL-6, and interferon (IFN)-*γ* were measured by ELISA using cytoscreen immunoassay kits (Peprotech, Rocky Hill, NJ, USA).

### 2.7. Assay of Splenocytes Proliferation

 Splenocytes (1 × 10^7^) from splenic tissue were suspended in RPMI-1640 medium with 10% fetal bovine serum, 2 mM glutamine, and 100 units of penicillin-streptomycin. Subsequently, the splenocytes were activated with phytohemagglutinin (PHA, Sigma) (20 *μ*g/mL) and lipopolysaccharide (LPS, Sigma) (20 *μ*g/mL). The cells were incubated at 37°C for 72 hrs. 3-(4,5)-Dimethylthiahiazol-2-yl-2,5-di-phenytetrazoliumromide (MTT, Sigma) were pulsed into each well during the final 6 hrs of incubation. The media was removed with a needle and syringe and dimethyl sulfoxide (DMSO, Kanto Chemical) was added to each well. Absorbance at 600 nm was measured.

### 2.8. Histological and Immunohistochemistry Assay

 Pancreas tissues taken from experimental animals were fixed in 10% neutral formalin, alcohol-dehydrated, paraffin-embedded and the section were cut at 4 *μ*m thickness. Sections were stained with hematoxylin and eosin (H & E). Adjacent secretions were also stained for insulin. Rabbit antirat insulin antibody (Cell Signaling, Danvers, MA, USA) was detected with an antirabbit avidin-biotinylated horseradish peroxidase complex (ABC) kit according to the manufacturer instructions (Vector, Burlingame, CA, USA). The degree of insulin expression in different groups was evaluated by software Image J (NIH, Maryland, MD, USA).

### 2.9. Statistical Analysis

 Data are expressed as the mean ± SD. The statistical significance was carried out using one-way analysis of variance test followed by Duncan's Multiple Range Test (SPSS statistical software package, version 12.0, SPSS, Chicago, IL, USA). A possibility of *P* value less than  .05 was considered as significantly different between means.

## 3. Results

### 3.1. Phenolic Compounds of Red Mold Dioscorea

**Figure 1:**
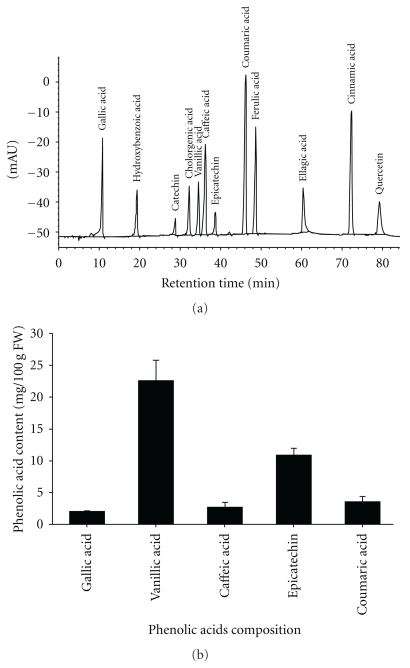
Chromatograms for phenolic acid. Phenolic acid standards (a). Phenolic acids composition of red mold dioscorea (b).

 Phenolic acids are aromatic secondary metabolites from plant and fungi [37] which have been associated with the color and nutritional and antioxidant properties of food [38].  Figure 1(a) shows chromatograms recorded at 280 nm for phenolic acid standards. The phenolic acid composition of red mold dioscorea (RMD) is given in Figure 1(b). The RMD sample contained 2.01 mg gallic acid/100 g FW, 22.58 mg vanillic acid/100 g FW, 2.67 mg caffeic acid/100 g FW, 10.87 mg epicatechin/100 g FW, and 3.54 mg coumaric/100 g FW. The contents of phenolic compounds in dioscorea were lower than *Monascus*-fermented dioscorea (<1 mg/100 g FW, data not shown). Phenolic compounds such as the natural antioxidants in plants may help to protect cells against the oxidative damage caused by neutralized free radicals [39] The present study shows that RMD containing a considerable amount of phenolic acid which may be useful in relation to diabetes involving oxidative stress.

### 3.2. Effect of Red Mold Dioscorea on Attenuating Symptoms of Diabetes

Before streptozotocin injection, the average level of blood glucose of experimental animals was 129.8 ± 10.8 mg/dL (data not show). After the experimental period, there was a significant increase in the levels of blood glucose, glycosylated hemoglobin (HbA1c) and free fatty acid (FFA). Concomitant decrease in the levels of insulin and total protein in the diabetic control group was shown in Table 1. As for the treatment groups (DM + 1X D, DM + 1X RMD, and DM + 5X RMD), the blood glucose and FFA levels were decreased, and the insulin level was increased. Levels of HbA1c and total protein were changed only in the group with 5X RMD. Rats in the diabetic control group had worse lipid profiles than the normal control ones (Table 2). Significant (*P* < .05) increases in total cholesterol (TC), triglyceride (TG), and low density lipoprotein (LDL) levels and decreases in high density lipoprotein (HDL) level were observed in the diabetic control group. The degraded lipid profiles of TC and LDL of the STZ-induced diabetic groups improved with treatment (DM + 1X D, DM + 0.5X RMD, DM + 1X RMD, and DM + 5X RMD). Groups DM + 1X RMD and DM + 5X RMD had a significant (*P* < .05) decrease in TG, TC, and LDL and an increase in HDL levels compared to the diabetic control group. Greater hypoglycemic and hypolipidemic effects in the STZ-induced diabetic rats were observed in the rats receiving 1X RMD and 5X RMD.

### 3.3. Effect of Red Mold Dioscorea on Organ Weight Change

 The liver weight per body weight in all STZ-treated groups was higher than in the normal control group (Table 3). Similarly, the kidney weight per body weight in all STZ-treated groups was higher than in the normal control group. Treating the STZ-induced rats with dioscorea and different doses of RMD had reduced the degree of renal hypertrophy at the termination of the eight-week treatment (Table 3).

### 3.4. Effect of Red Mold Dioscorea on Antioxidant Enzymes in Pancreas

**Figure 2:**
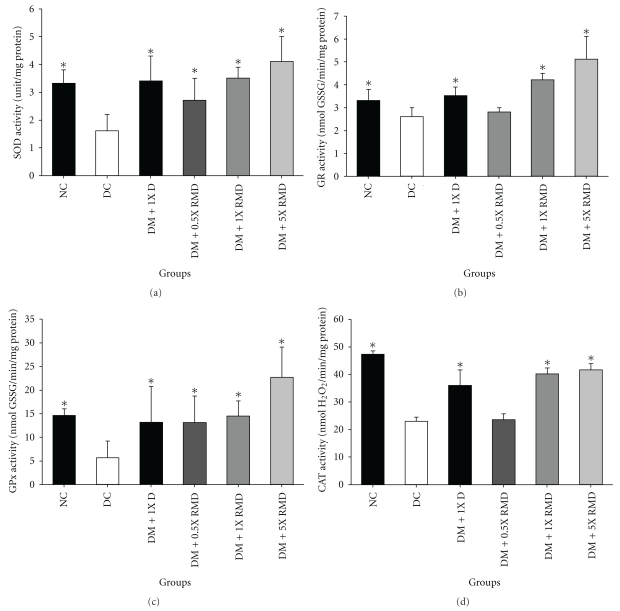
Effect of red mold dioscorea on antioxidant enzyme activity in the pancreas. Superoxide dismutase (SOD) (a). Glutathione reductase (GR) (b). Glutathione peroxidase (GPx) (c). Catalase (CAT) (d). NC: normal control; DC: diabetic control; DM: diabetes mellitus; D: dioscorea; RMD: red mold dioscorea. Data are presented as the means ± SD (*n* = 6). *P < .05 versus the diabetic control.

 An increase in the production of tissue-damaging ROS by glucose autoxidation and nonenzymatic protein glycosylation was observed in diabetes [40]. Using antioxidants to protect the oxidative damage to the pancreatic cells has a therapeutic effect on diabetes. The ROS level of the pancreas was generated in abundance by the STZ-induced diabetic group compared to the normal group (Figure 2). Groups with treatment (1X D, 1X RMD and 5X RMD) provided a significant (*P* < .05) reduction of ROS in the pancreas (data not show). The activities of enzymatic antioxidants (GR, GPx, SOD, and CAT) were constantly and significantly (*P* < .05) decreased in the STZ-induced diabetic group compared to the normal control group. The GPx activities of the pancreas were increased in all of the treatment groups. The GR, CAT, and SOD activities of the pancreas were increased in the groups of DM + 1X D, DM + 1X RMD, and DM + 5X RMD. These results indicated an enhancement of the enzyme activity when treating the 1X RMD and 5X RMD.

### 3.5. Effect of Red Mold Dioscorea on Splenocytes Proliferation

**Figure 3:**
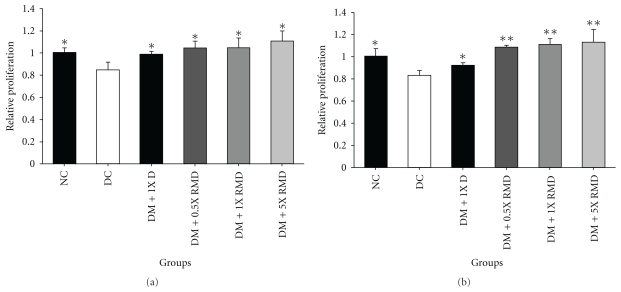
Relative proliferation of splenocyte from rats with red mold dioscorea treatment. Splenocytes were stimulated with PHA (a) and LPS (b). NC: normal control; DC: diabetic control; DM: diabetes mellitus; D: dioscorea; RMD: red mold dioscorea. *P < .05 versus the diabetic control.

 Lymphocyte plays an important role in the immune function and ensures a functional repertoire of mature B and T cells [41, 42]. The mitogen (PHA and LPS) response of splenocytes showed a significant (*P* < .05) decrease in the diabetic control rats compared to normal control rats (Figure 3). The groups increased the proliferation activity of splenocytes with treatment. The results suggest that dioscorea and RMD have beneficial effects on improving the state of a decreased cellular immune function in STZ-induced diabetic rats.

### 3.6. Effect of Red Mold Dioscorea on Cytokines Production

 The fact that diabetes mellitus shares similarities with inflammatory diseases is considered to play a role in diabetic complications [43]. The cytokine levels of TNF-*α*, IL-1*β*, and IL-6 were increased in the patients with diabetes and diabetic rats [43, 44]. In our study, RMD treatments reduced the TNF-*α* level in the pancreas and kidneys (Table 4). IFN-*γ* and IL-6 levels in the kidneys were decreased in all of the treated groups, and the level of IL-1*β* in the pancreas was decreased only in DM + 1X RMD, and DM + 5X RMD treated rats. Spleen expression of IFN-*γ* level in the DM + 5X RMD group was significantly (*P* < .05) lower than in the diabetic control group. Spleen expression of IL-1*β* level in the diabetic control group was significantly (*P* < .05) higher than the DM + 1X D, DM + 1X RMD and DM + 5X RMD groups. The level of IL-2 was increased in the pancreas, kidney and spleen.

### 3.7. Immunohistochemical Evaluation on Pancreas

**Figure 4:**
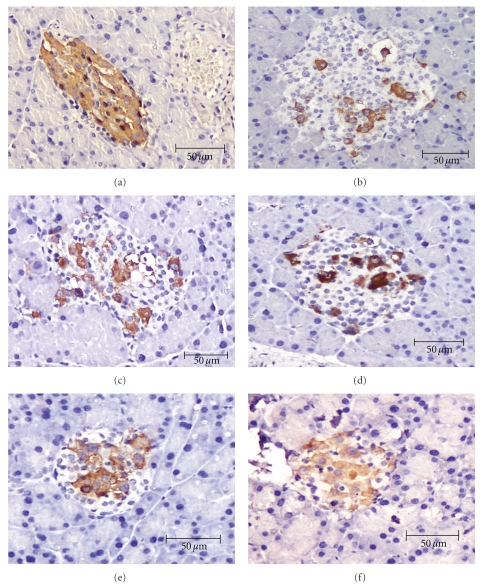
Immunohistochemical evaluation on pancreas (400x). Normal control (a). Diabetic control (b). Diabetic + 1X D (dioscorea) (c). Diabetic + 0.5X RMD (red mold dioscorea) (d). Diabetic + 1X RMD (red mold dioscorea) (e). Diabetic + 5X RMD (red mold dioscorea) (f).

**Figure 5:**
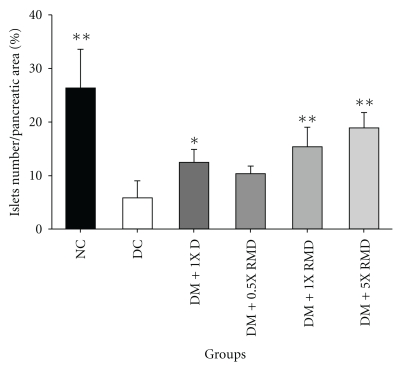
Comparative evaluation of the expression of insulin immunoreactivity in pancreas. NC: normal control; DC: diabetic control; DM: diabetes mellitus; D: dioscorea; RMD: red mold dioscorea. Data are presented as the means ± SD (*n* = 6). *P < .05 versus the diabetic control.

 An immunohistochemical analysis of the pancreatic tissues is shown in Figure 4. In the normal control group, the islets showed the normal structure by insulin-secreting *β*-cells (Figure 4(a)). The islets of the pancreatic of the diabetic control group, the insulin immunoreactivity and the number of immunoreactivity of *β*-cells were decreased (Figure 4(b)). The diabetic rats given 0.5X RMD showed no difference from the diabetic control rats (Figure 4(d)). In contrast, the diabetic rats given 1X D, 1X RMD, and 5X RMD resulted in a significant (*P* < .05) increase in the insulin immunoreactivity, and a number of *β*-cells were observed in the pancreatic islets compared with diabetic control rats (Figures 4(c), 4(e), and 4(f)). The result of the image analysis of the insulin immunoreactivity of *β*-cells is shown in Figure 5. After being treated with 1X D, 1X RMD, and 5X RMD, the insulin immunoreactivity was relatively more frequent than the diabetic control group, especially when treated with 1X RMD and 5X RMD (Figure 5). The results indicate that RMD have a cytoprotective effect in pancreatic tissues.

## 4. Discussion

Hyperglycemia, an indicator for abnormal glucose homeostasis, is related to diabetes, incidences of obesity, and cardiovascular risk, inducing peripheral insulin resistance [45]. Recent studies indicate that hyperglycemia-induced diabetic complications are likely from oxidative dysfunction and inflammatory effect [5, 6]. The *Monascus* species can produce multifunctional compounds; the composition of incubation substrates is an important factor for the production of secondary metabolites [46] and the growth of *Monascus *spp. In our previous study, RMD had greater hypolipidemic, antiatherosclerotic, and antihypertension effects than red mold rice (RMR) and unfermented dioscorea [20, 47]. RMR contains various antioxidants, including dimerumic acid, organic acid, tannin, and phenolic content [20]. Our present study provides *in vivo* data to elucidate the antioxidative and anti-inflammatory effects of RMD in diabetic rats. In the HPLC assay, we found that RMD has different phenolic acid compounds, such as gallic acid, vanillic acid, caffeic acid, coumaric acid, and epicatechin (Figure 1(b)). Those phenolic acids, as antioxidants, have several biological activities to reduce lipid peroxyl radicals, inhibit lipid peroxidation, and protect our cells against oxidative damage [48, 49]. 

Elevated levels of free fatty acid have been inhibiting insulin secretion by mitochondrial oxidation in the establishment of insulin resistance in diabetes mellitus [50]. Plasma FFA levels are commonly associated with impaired insulin-mediated glucose uptake in related tissues, and coexist with type 2 diabetes and obesity [51]. Plasma total protein can be used to test the effects of treatment in diabetes and nephrotic syndrome [52]. The current study shows a significant (*P* < .05) decrease in blood glucose and the FFA levels of STZ-induced diabetic rats with RMD treatment (Table 1). The insulin level was increased in all groups with treatment. Low total protein can result from protein loss such as nephrosis, glomerulonephritis, chronic liver disease, and hemorrhage. Levels of HbA1c and total protein were decreased and increased, respectively, only in DM + 5X RMD rats. This result provides direct proof of the antihyperglycemic and anti-inflammatory-like functions of RMD.

Many studies have attempted to determine if antioxidants can actually prevent the oxidation of LDL and slow the progression of atherosclerosis [53]. Other studies suggest that diabetic individuals may have an increased susceptibility to LDL oxidation [53]. High triglyceride levels in the blood tend to coexist with low levels of high-density lipoprotein (HDL), contributing to a condition called diabetic dyslipidemia or hypertriglyceridemia [54]. The cholesterol and triglycerides cause a risk of heart disease, which should be as tightly controlled as possible in diabetes mellitus [55]. The present study demonstrates that dioscorea and RMD can significantly lower serum TC and LDL levels (*P* < .05) (Table 2). Levels of TG and HDL were decreased and increased respectively only in DM + 1X RMD and DM + 5X RMD rats.

Renal hypertrophy is an important early manifestation of both experimental and human diabetes, although the metabolic events responsible for its development are not yet completely understood. The kidneys are enlarged in diabetes mellitus when they have microalbuminuria or overt levels of albumin excretion [56]. A raised level of albumin in the urine is a typical indicator that the kidneys have been injured by diabetes [57]. Excess body weight has an adverse effect on the liver, and can increase the risk of the development of metabolic symptoms [58]. The indices of kidney weight/body weight and liver weight/body weight ratio are shown in Table 3. The liver weight was increased in all of the STZ-induced diabetic rats, although the increases tended to be less in the rats treated with dioscorea and RMD. The kidney weight in diabetic control rats was significantly (*P* < .001) greater than it was in normal control rats. The kidneys of diabetic rats with treatment were significantly (*P* < .05) lower than they were in diabetic control rats, especially those treated with 5X RMD. Although STZ-induced diabetic rats showed liver and kidney hypertrophy, the measurement of other indicators such as histopathology is needed to be further investigated.

A diabetogenic effect of STZ was observed due to the excess production of ROS, which led to cytotoxicity in pancreatic *β*-cells [59]. Oxidative stress may be an important factor in the pathogenesis of different diabetic complications [60]. Reduced activities of GR, GPx, CAT, and SOD in pancreas of diabetic control rats were observed in our study (Figure 2). Treatment with dioscorea and RMD reversed this change. Thus, it is possible that dioscorea and RMD prevented ROS-induced damage to the elevation of antioxidant enzymes activities. According to the results, RMD may have an antioxidative effect against the pathological alterations caused by ROS.

In diabetes, the suppressed proliferation potential of diabetic T lymphocytes with mitogen stimulated was achieved by a decreased expression of adenosine kinase [60], and decreased IL-2 production by mitogen-stimulated diabetic T cells has been reported [61]. These mechanisms are still unclear as to why these cells display an impaired proliferative response to primary antigens. In our study, similar results have been observed, in that the mitogen response of splenocytes showed a significant (*P* < .05) decrease in the diabetic control rats, and rats with treatment had an increased proliferation activity of splenocytes (Figure 3). The dioscorea and RMD have beneficial effects on enhancing spleen cells viability and improving the state of decreased cellular immune function in diabetic rats. Our present study found that RMD treatments reduced IFN-*γ*, IL-1*β*, IL-6, and TNF-*α* levels in the pancreas, the kidney, and the spleen (Table 4). The level of IL-2 was increased in the pancreas, kidney and spleen. The level of TNF-*α* was no different in the spleen. The levels of IL-6 and IL-1*β* were no different in the pancreas and the kidney, respectively. The first factor at the crossroads of inflammation and metabolic disease was TNF-*α*. The production of excess TNF-*α* in tissue was due to insulin resistance [12]. The islet inflammatory process in the diabetic rats showed an increased islet expression of IL-1*β*, IL-6, and TNF-*α* [62]. The increased production of IL-6 and IFN-*γ* by STZ-induced diabetic rats was identified as autoimmune diabetes [63]. The kidney macrophages can produce a variety of substances which can promote renal injury, such as nitric oxide, ROS, IFN-*γ*, and IL-1, and TNF-*α* resulted in renal injury in diabetic nephropathy [64]. IL-2 has a well-established function to promote T-cell proliferation and low production in STZ-induced diabetic rats [65]. These results support the fact that RMD is a potent agent against diabetes-associated inflammatory injury via inhibiting inflammatory cytokines production and enhancing IL-2 expression.

**Figure 6:**
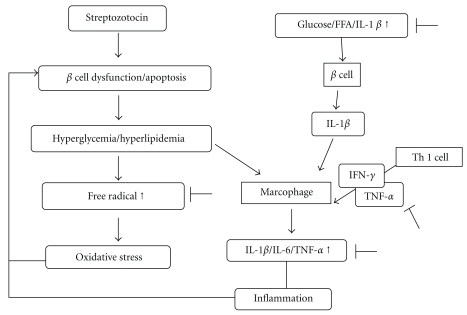
The hypothetical diagram of prevention approach of red mold dioscorea with diabetic oxidative stress and inflammatory response.

The beneficial effects of RMD observed in the present study were the maintenance of glucose homeostasis, the prevention of oxidative stress and inflammatory activities, and the protection of pancreatic *β*-cells. A hypothetical diagram of the preventative approach of red mold fermented products was presented (Figure 6). The role of RMD in controlling diabetic-reactive inflammation is unclear, and further study is necessary on RMD to determine the interaction between it and diabetes-associated symptoms.

## 5. Conclusion

The pathogenesis of diabetes and diabetes complications is complex. RMD, a novel red mold fermented product, has a beneficial effect in the STZ-induced diabetic animal model. The present investigation shows that RMD possesses several treatment-oriented properties, including the control of hyperglycemia, antioxidant effects, pancreatic *β*-cell protection, and anti-inflammatory effects. Considering these observations, it appears that RMD may be a useful adjunct supplement to delay the development of diabetes and diabetes complications. However, more work is necessary to further elucidate the role of RMD, particularly looking at the underlying mechanism of treatment.

## Figures and Tables

**Table 1:** Effect of red mold dioscorea on biochemistry parameters in STZ-induced diabetic rats.

Groups	Glucose	Insulin	HbA1c	FFA	Total protein
	mg/dL	mmol/mL	%	mmol/L	g/dL
Normal control (NC)	143.6 ± 8.5	153.7 ± 11.2	4.2 ± 0.2	0.9 ± 0.1	6.7 ± 0.2
Diabetic control (DC)	438.3 ± 25.5	48.2 ± 9.1	7.5 ± 0.2	1.3 ± 0.8	5.4 ± 0.4
DM + 1X D	383.8 ± 39.2*	80.5 ± 9.6*	7.0 ± 0.7	0.8 ± 0.2*	5.8 ± 0.6
DM + 0.5X RMD	392.9 ± 22.6	72.5 ± 9.5*	7.3 ± 0.4	0.9 ± 0.1	5.8 ± 0.4
DM + 1X RMD	380.6 ± 41.0*	83.9 ± 12.5*	7.2 ± 0.5	0.8 ± 0.4*	5.9 ± 0.5
DM + 5X RMD	370.2 ± 35.7*	93.3 ± 15.8*	6.1 ± 1.1*	0.7 ± 0.2*	6.5 ± 1.0*

FFA: free fatty acid; HbA1c: glycosylated hemoglobin; DM: diabetes mellitus; D: dioscorea; RMD: red mold dioscorea. Values are expressed as mean ± SD (*n* = 6). *P < .05 versus the diabetic control.

**Table 2:** Effect of red mold dioscorea on serum lipid profile in STZ-induced diabetic rats.

Groups	TG	TC	HDL-C	LDL-C	TC/HDL
	mg/dL				
Normal control (NC)	89.4 ± 13.3	74.8 ± 9.2	58.2 ± 5.8	10.5 ± 3.0	1.29 ± 0.02
Diabetic control (DC)	332.0 ± 49.0	127.7 ± 38.0	48.1 ± 8.1	18.2 ± 6.7	2.65 ± 0.04
DM + 1X D	268.6 ± 84.1	78.2 ± 20.1*	48.6 ± 8.6	9.2 ± 3.6*	1.61 ± 0.05*
DM + 0.5X RMD	270.4 ± 58.6	89.7 ± 27.1*	49.3 ± 8.1	9.4 ± 3.7*	1.82 ± 0.09*
DM + 1X RMD	217.8 ± 51.2*	83.3 ± 19.5*	52.2 ± 5.7*	10.1 ± 1.7*	1.59 ± 0.06*
DM + 5X RMD	193.4 ± 37.5*	78.3 ± 17.4*	56.0 ± 5.2*	9.9 ± 3.4*	1.40 ± 0.04*

TG: triglyceride; TC: total cholesterol; HDL-C: high density lipoprotein cholesterol; LDL-C: low density lipoprotein cholesterol; DM: diabetes mellitus; D: dioscorea; RMD: red mold dioscorea. Values are expressed as mean ± SD (*n* = 6). *P < .05 versus the diabetic control.

**Table 3:** Effect of red mold dioscorea on tissue weight of STZ-induced diabetic rats.

Groups	Whole body weight	% of body weight			
	g	Heart	Lung	Liver	Kidney
Normal control (NC)	404.8 ± 44.5	0.39 ± 0.03	0.57 ± 0.11	3.47 ± 0.18	0.94 ± 0.04
Diabetic control (DC)	294.6 ± 73.3	0.47 ± 0.18	0.62 ± 0.17	4.71 ± 1.44	1.43 ± 0.47
DM + 1X D	291.9 ± 27.7	0.47 ± 0.08	0.56 ± 0.07	4.50 ± 0.47*	1.21 ± 0.47*
DM + 0.5X RMD	280.6 ± 38.8	0.43 ± 0.06	0.57 ± 0.07	4.18 ± 0.34*	1.16 ± 0.11*
DM + 1X RMD	289.1 ± 58.2	0.41 ± 0.05	0.59 ± 0.03	4.20 ± 0.58*	1.14 ± 0.18*
DM + 5X RMD	329.5 ± 73.2	0.39 ± 0.10	0.58 ± 0.17	4.06 ± 1.23*	1.02 ± 0.16*

DM: diabetes mellitus; D: dioscorea; RMD: red mold dioscorea. Values are expressed as mean ± SD (*n* = 6). *P < .05 versus the diabetic control.

**Table 4:** Effect of red mold dioscorea on inflammatory cytokines in STZ-induced diabetic rats.

Pancreas						
Cytokine type (pg/mL)	NC	DC	DM + 1X D	DM + 0.5X RMD	DM + 1X RMD	DM + 5X RMD
TNF-*α*	52.2 ± 8.4	158.2 ± 20.5	114.1 ± 14.4**	116.0 ± 17.1**	108.8 ± 19.1**	97.6 ± 23.2**
IL-1*β*	19.5 ± 5.9	40.9 ± 9.8	33.3 ± 6.4	34.3 ± 5.6	31.5 ± 3.8*	29.8 ± 9.8**
IL-2	119.5 ± 18.0	56.7 ± 13.8	136.7 ± 32.4*	129.4 ± 27.2*	148.0 ± 9.7*	140.5 ± 12.7*
IL-6	38.4 ± 7.9	95.5 ± 16.0	83.3 ± 11.3	84.9 ± 10.7	80.4 ± 10.3	77.2 ± 10.3

Kidney						

IFN-*γ*	10.6 ± 2.0	30.4 ± 3.9	23.2 ± 3.5*	23.2 ± 3.9*	21.6 ± 2.6*	21.1 ± 5.3*
TNF-*α*	28.8 ± 6.4	147.7 ± 13.5	119.0 ± 10.6**	122.6 ± 9.3**	119.4 ± 22.8**	115.6 ± 9.2**
IL-6	53.2 ± 8.5	186.6 ± 18.9	152.7 ± 13.2**	156.3 ± 28.0**	147.0 ± 20.5**	140.6 ± 10.8**
IL-1*β*	9.7 ± 1.6	16.2 ± 2.9	15.7 ± 2.2	15.7 ± 2.2	15.6 ± 1.3	15.1 ± 1.5
IL-2	55.8 ± 9.0	44.9 ± 8.8	75.0 ± 19.0*	69.1 ± 15.7*	80.4 ± 23.3*	82.9 ± 18.3**

Spleen						

TNF-*α*	65.3 ± 16.1	131.6 ± 24.6	124.3 ± 56.9	123.0 ± 8.7	123.2 ± 25.1	122.4 ± 27.2
IFN-*γ*	14.0 ± 2.1	42.7 ± 7.0	36.6 ± 5.6	39.4 ± 7.8	36.2 ± 9.4	32.8 ± 5.2*
IL-1*β*	25.4 ± 5.1	36.9 ± 5.1	32.0 ± 2.9*	32.9 ± 5.5	30.7 ± 3.6*	30.1 ± 2.0*
IL-2	33.8 ± 9.5	13.8 ± 2.5	23.0 ± 5.4*	22.9 ± 6.1*	25.3 ± 2.7*	27.6 ± 4.6*

NC: normal control; DC: diabetic control; DM: diabetes mellitus; D: dioscorea; RMD: red mold dioscorea. Values are expressed as mean ± SD (*n* = 6). *P < .05; **P < .01 versus the diabetic control.
